# External Quality Assessment of Molecular Detection of Ebola Virus in China

**DOI:** 10.1371/journal.pone.0132659

**Published:** 2015-07-15

**Authors:** Guojing Wang, Yu Sun, Kuo Zhang, Tingting Jia, Mingju Hao, Dong Zhang, Le Chang, Lei Zhang, Rui Zhang, Guigao Lin, Rongxue Peng, Jinming Li

**Affiliations:** 1 National Center for Clinical Laboratories, Beijing Hospital of National Health and Family Planning Commission, Beijing, People’s Republic of China; 2 Graduate School, Peking Union Medical College, Chinese Academy of Medical Sciences, Beijing, People’s Republic of China; 3 Fifth School of Clinical Medicine, Peking University, Beijing, People’s Republic of China; Division of Clinical Research, UNITED STATES

## Abstract

In 2014, Ebola hemorrhagic fever broke out in West Africa. As contact between China and West Africa is frequent, the possibility that Ebola virus would enter China was high. Thus, an external assessment of the quality of Ebola virus detection was organized by the National Center for Clinical Laboratories in China. Virus-like particles encapsulating known sequences of epidemic strains of Ebola virus from 2014 were prepared as positive quality controls. The sample panel, which was composed of seven positive and three negative samples, was dispatched to 19 laboratories participating in this assessment of Ebola virus detection. Accurate detection was reported at 14 of the 19 participating laboratories, with a sensitivity of 91.43% and a specificity of 100%. Four participants (21.05%) reported false-negative results and were classified as “acceptable.” One participant (5.26%) did not detect any positive samples and was thus classified as “improvable.” Based on the results returned, the ability to detect weakly positive Ebola specimens should be improved. Furthermore, commercial assays and the standard primers offered by the Chinese Centers for Disease Control and Prevention were found to be most accurate and dependable for Ebola detection. A two-target detection approach is recommended for Ebola screening; this approach could reduce the probability of false-negative results. Additionally, standardization of operations and punctual adjustment of instruments are necessary for the control and prevention of Ebola virus.

## Introduction

From 2013 to 2015, the outbreak of Ebola hemorrhagic fever (EHF) caused over 22,000 cases of infection, including nearly 9,000 deaths (as of February 4, 2015) [1] in West Africa. The Ebola virus has an 18–19 kb, non-segmented, single-stranded negative-RNA genome that encodes seven structural proteins (nucleoprotein (NP), virion structural protein (VP)35, VP40, glycoprotein (GP), VP30, VP24, and RNA-dependent RNA polymerase (L)) [2]. Virological research and analysis recently identified the epidemic agent as Zaire Ebola virus (EBOV) [3]. The characteristics of infection with EBOV are high mortality [4] and catastrophic clinical syndromes. Owing to a lack of effective intervention and treatment, early diagnosis and isolation are the primary methods to resist EBOV infection [5]. For early detection of EBOV [6], real-time reverse transcription polymerase chain reaction (RT-PCR) is the method most commonly and frequently used [7, 8].

On August 8, 2014, the World Health Organization (WHO) announced the Ebola outbreak in West Africa as a global public health event. In fact, although the EBOV epidemic was concentrated in West Africa, cases of infection were also reported in other areas, such as the USA, Spain, and Scotland. Although EBOV showed a sporadic distribution, the possibility of Ebola infections appearing outside West Africa could not be neglected. China is one of the countries in closest contact with West Africa, and a large number of people entered and exited China, especially in harbor cities, from the areas affected by EBOV by airline. As a result, there was a risk that EBOV would enter China. Screening of high-risk populations from EBOV-affected regions was therefore essential not only for control and prevention of Ebola in Chinese native, but also as part of the global task of fighting the plague.

Hence, it was important to evaluate the EBOV detection capability in key port cities in China, along with the reagents employed by the testing agencies. Therefore, the National Center for Clinical Laboratories (NCCL) organized an external quality assessment (EQA) of EBOV detection in China.

## Materials and Methods

### Preparation and composition of panel

Nucleotide sequences of the epidemic strain of Zaire Ebola virus from West Africa were obtained from GenBank (KJ660346, KJ660347, and KJ660348) [3]. Synthesis of the target segments of the EBOV genome was outsourced to Thermo Fisher Scientific Inc. (Beijing, China), including the full sequence of the 3’-untranslated region (UTR) and the NP fragment, and portions of the sequence of the GP, L, and 5’-UTR regions. Synthesis of the target sequences was divided into two parts: the first encompassed nearly 2400 bp (the 3′-UTR and a large part of the NP region), and the other encompassed approximately 2250 bp (the remaining part of the NP region, and selected parts of the GP, L, and 5′-UTR regions). The primers used are listed in S1 Table. The two synthesized fragments were subcloned into the pACYC-MS2 vector at the KpnI/PacI and BglII/KpnI restriction sites, respectively, to form the recombinant plasmids pACYC-MS2-NP and pACYC-MS2-GP/L. The expression and purification of the two types of MS2 virus-like particles (VLPs) was performed according to previously published protocols [9, 10].

Two different types of EBOV MS2 VLPs were used as positive samples. The sample panel was comprised of three negative and seven positive samples, ranging from 8 × 10^2^ to 5 × 10^7^ copies/mL, in accordance with previously reported clinical outcomes [11]. Three negative samples and dilution buffer for positive ones were composed of 30% normal human serum and 70% normal saline.

Of the seven positive samples, No. 1402 contained the highest concentration of EBOV VLPs (Table 1). No. 1408 also contained hepatitis C virus (HCV) and measles virus (MV) VLPs, to verify whether EBOV detection is impacted by the presence of other viruses. Repeatability was evaluated by the use of two replicate specimens (No. 1403 and No. 1407). No.1410 was prepared to evaluate the ability to detect slightly positive samples. Additionally, one specimen (No. 1405) contained only a very weak concentration of the NP fragment and a moderate concentration of the GP/L EBOV fragment. Prior to distribution, each sample panel was confirmed using three commercial kits manufactured in China (Daan Gene Co., Ltd., Guangzhou; ZJ Bio-Tech Co., Ltd., Shanghai; and Puruikang Bio-Tech Co., Ltd., Shenzhen).

**Table 1:** Composition and results of the sample panel.

Sample No.	Concn of armored RNAs in samples (copies/ml)		Classification	No. of correct/total No. tested (%)
	NP	GP/L		
**EBOV-1402**	10^7^	5×10^7^	Strong positive	19/20 (95%)
**EBOV-1408**	5×10^6^	8×10^6^	Positive(HCV and MV VLP)	19/20 (95%)
**EBOV-1404**	10^6^	5×10^6^	Positive	19/20 (95%)
**EBOV-1403**	10^5^	5×10^5^	Positive	19/20 (95%)
**EBOV-1407**	10^5^	5×10^5^	Positive	19/20 (95%)
**EBOV-1410**	8×10^3^	2×10^3^	Slight positive	18/20 (90%)
**EBOV-1405**	8×10^2^	10^6^	Slight positive(NP)	15/20 (75%)
			Positive(GP/L)	
**EBOV-1401**	-	-	Negative	20/20 (100%)
**EBOV-1406**	-	-	Negative	20/20 (100%)
**EBOV-1409**	-	-	Negative	20/20 (100%)

One of nineteen participators reported 2 results (both NP and GP sites).

### Stability assessment

The stability of two types of EBOV MS2 VLPs with the dilution buffer of 30% normal human serum and 70% normal saline was evaluated at different times and temperatures. Two different concentrations of VLPs were used: 10^5^ copies/mL and 10^3^ copies/mL. For each of the concentrations, 15 samples were included. Three samples were stored at -80°C as a control. The remaining 12 samples were divided into three groups and incubated at different temperatures: room temperature, 4°C, and -20°C. In each group, two samples were removed and stored at -80°C for 10 days or 20 days. After 20 days, all 15 samples for each concentration were simultaneously assessed using EBOV commercial assays (real-time RT-PCR kit, Daan Gene Co., Ltd., Guangzhou, China).

The stability of the samples during distribution and transport was monitored. A sample panel was sent unmarked by mail to a deliberately non-existent destination and returned to NCCL. The returned sample panel was assessed simultaneously with another that had been stored at -80°C, using an EBOV RT-PCR assay manufactured by Daan Gene Co., Ltd. In all assays, stability was designated as a reduction of < 0.5 log_10_ copies/mL, compared with the results for samples stored at -80°C.

### Organization and Transport

**Fig 1:**
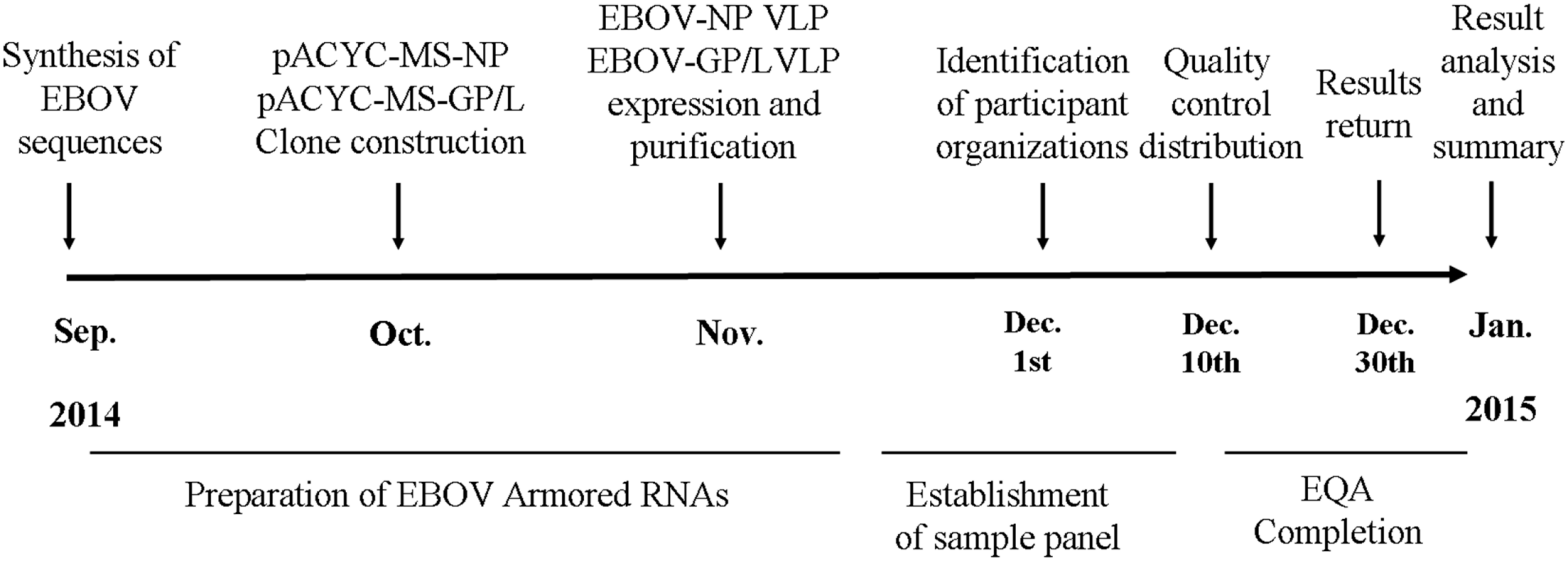
A schematic map of the experimental procedure.

All qualified laboratories, institutions for inspection and quarantine, and manufacturers of kits for EBOV detection in mainland China were invited to participate in this investigation in November 2014. All participating organizations were identified by early December (Fig 1). All samples were distributed by NCCL on December 10, 2014. Qualitative results, threshold cycle (Ct) values, RNA extraction and detection methods, and primer sources were all required to be reported to NCCL by December 30, 2014. The full EQA process and related time points are shown in Fig 1.

### Appraisal and statistical analysis

The results were classified into three categories: competent (100% correct responses), acceptable (≤ 2 incorrect results), or improvable (> 2 incorrect results). All data were analyzed using SPSS, version 19.0; the variances of different groups were compared using Fisher’s exact test or Pearson’s chi-square test, when appropriate.

## Results

### Preparation, stability, and evaluation of the sample panel

Construction of the recombinant plasmids pACYC-MS2-NP and pACYC-MS2-GP/L was successful. A series of experiments was conducted to verify the effects of VLP expression and packaging capability. Target protein purification in approximately 60 minutes (S1A Fig) and observation of a 14-kD band following sodium dodecyl sulfate polyacrylamide gel electrophoresis (SDS-PAGE) (S1B Fig) were preliminary evidence for successful VLP expression. After digestion with RNase A and DNase I at 37°C, observation of a single band over 1 kb in length in 1% agarose gel electrophoresis (S1C Fig) indicated the stability of the EBOV VLPs. RT-PCR results showing a target band between 2–2.5 kb (S1D Fig) served as further evidence of successful encapsulation of the target EBOV segments in VLPs after extraction of nucleotides. The concentrations of the two types of EBOV VLPs were each approximately 5 × 10^11^ copies/mL.

In the stability evaluation, the variations of two concentrations of samples in different temperatures were presented in form of mean Ct±standard deviation (SD), which indicated in S2 Table. Samples stored at different conditions showed less than 0.5 log_10_ variations in concentration, compared with samples incubated at -80°C. Meanwhile, the comparisons of sample panels saved in -80°C and from blind mail were showed in S2 Fig. No statistical difference was observed between test results for sample panels sent unmarked through the mail and those stored at -80°C.

The sample panel was verified before distribution using the three commercial kits mentioned above; the results were concordant with the results shown in Table 1. The results for No. 1403 and No. 1407 were similar. No obvious interference of HCV or MV with EBOV detection was observed for No. 1408. The slightly positive samples (No. 1405 and No. 1410) could be detected.

### Participating agencies and methodologies

A total of 19 agencies took part in the assessment of the quality of molecular detection of EBOV, including three provincial Centers for Disease Control and Prevention (CDC) (Shanghai, Zhejiang, and Jiangsu), six Entry-Exit Inspection and Quarantine agencies in port cities (Fujian, Hainan, Hebei, Yunnan, Zhejiang, and Zhuhai), one public health platform in Shanghai, one fixed point hospital qualified for EBOV detection(Chongqing), and eight manufacturers of EBOV detection kits(Daan Gene Co., Ltd., ZJ Bio-Tech Co., Ltd., HuaDa BioTech Co., Ltd., KeHua Bio-Engineering Co., Ltd., TianLong Bio-Tech Co., Ltd., BioPerfectus Technologies, Sansure Bio-Tech Co., Ltd., and Puruikang Bio-Tech Co., Ltd.). All participating organizations had certification in China for EBOV screening and diagnosis.

At the 19 participating agencies, both magnetic bead and spin column methods were used for nucleic acid extraction. The paramagnetic particle method was preferred by nine organizations, the spin column method by the other ten (Table 2). All of the laboratories that used the spin column method used manual extraction, whereas 2/3 of the agencies using the paramagnetic particle method used instruments. For detection, only one agency used RT-PCR and agarose gel electrophoresis, whereas most chose real-time PCR. Among the 18 agencies that selected quantitative real-time PCR, 83.33% used commercial kits. The specified primers offered by the national CDC [12] (Table 3), one of which returned results for both the NP and GP sites, were preferred by two provincial CDCs. Additionally, one agency used a real-time PCR assay developed in-house for EBOV diagnosis.

**Table 2:** Performance of 19 laboratories participating in the EQA for EBOV.

Laboratory No.	Extraction kit^a^	Extraction method	Real time RT-PCR assay^b^	No. of correct sample/total no. of samples (%)	Classification
1	QIAamp	Spin column	A	100	Competent
4	QIAamp	Spin column	A	100	Competent
5	QIA EZ1	Magnetic bead	A	100	Competent
8	QIAamp	Spin column	A	100	Competent
12	Daan gene	Magnetic bead	A	100	Competent
6	QIAamp	Spin column	B	100	Competent
15	ZJ Bio-Tech	Magnetic bead	B	100	Competent
9	Life	Magnetic bead	B	80	Acceptable
18	Puruikang	Magnetic bead	C	100	Competent
13	Tiangen	Spin column	D	100	Competent
17	Tianlong	Magnetic bead	E	90	Acceptable
16	BioPerfectus	Spin column	F	90	Acceptable
19	Sansure	Magnetic bead	G	90	Acceptable
14	Kehua	Magnetic bead	H	100	Competent
7	QIAamp	Spin column	I	100	Competent
2	RNeasy	Magnetic bead	In-house (CDC)	100	Competent
3	QIAamp	Spin column	In-house (CDC)	100	Competent
10	Tianze	Spin column	In-house (RT-PCR)	100	Competent
11	QIAamp	Spin column	In-house (real time)	30	Improvable

^a^: QIAamp, QIAamp viral RNA minikit (Qiagen); QIA EZ1, Qiagen EZ1 virus mini kit (Qiagen); Daan gene, Daan gene viral RNA kit; RNeasy, RNeasy minikit (Qiagen); ZJ Bio-Tech, ZJ Bio-Tech viral RNA kit; Life, ABI Life Technologies AM 1836 MagMAX-96 ViralRNA Isolation Kit; Puruikang, Puruikang viral RNA kit; Tiangen, Tiangen viral RNA kit; Tianlong, Tianlong viral RNA kit; Sansure, Sansure viral RNA kit; Kehua, Kehua viral RNA kit; Tianze, Tianze RNAout kit.

^b^: A: Daan Gene (Daan Gene Co., Ltd., Guangzhou, China); B: ZJ Bio-Tech (ZJ Bio-Tech Co., Ltd., Shanghai, China); C: Puruikang (Puruikang Bio-Tech Co., Ltd., Shenzhen, China); D: Huada BioTech (HuaDa BioTech Co., Ltd., Guangzhou, China); E: Tianlong (TianLong Bio-Tech Co., Ltd., Suzhou, China); F: BioPerfectus (BioPerfectus Technologies, Jiangsu, China); G: Sansure (Sansure Bio-Tech Co., Ltd., Hunan, China); H: Kehua (ShangHai KeHua Bio- Engineering Co., Ltd., Shanghai, China); I: NIFDC (National Institutes for Food and Drug Control, Beijing, China); In-house(CDC): in-house conventional real time RT-PCR assay for EBOV, primers offered by China CDC, including NP and GP sites; In-house(RT-PCR): ordinary RT-PCR and agarose gel electrophoresis method; In-house (real time): in-house real time RT-PCR assay for EBOV, primers designed by themselves.

**Table 3:** The primers and probes provided by Chinese CDC.

primers and probes	Sequences	Fluorescent labels
ZEBONP-F	CGCCGAGTCTCACTGAATCTG	
ZEBONP-R	AGTTGGCAAATTTCTTCAAGATTGT	FAM/BHQ-1
ZEBONP-P	CGGCAAAGAGTCATCCCAGTGTATCAAGTA	
ZEBOGP-F	TGGGCTGAAAAYTGCTACAATC	
ZEBOGP-R	CTTTGTGMACATASCGGCAC	FAM/BHQ-1
ZEBOGP-P	CTACCAGCAGCGCCAGACGG	

### Laboratory performance

The performance of the 19 participating agencies is shown in Table 2. The performance of 14 of the 19 agencies (73.68%) was found to be competent (100% correct responses). Three participants (15.79%) reported one false-negative and one (5.26%) reported two false-negatives; these were classified as acceptable (21.05%) according to the criteria stated above. One participant (5.26%) was unable to obtain any positive results in this assessment, and was thus included in the improvable group.

In all, among the 200 results returned, 12 (6%) were incorrect, including 8.57% false-negative results and no false-positives. Seven of these incorrect results were returned by one agency. No. 1405 was not detected by five different laboratories, which reported very weakly positive results for the NP (8 × 10^2^ copies/mL) fragment. The pair of duplicate specimens (No. 1403 and No. 1407) with similar Ct values was reported correctly by 18 institutions. No. 1410 (NP: 8 × 10^3^ copies/mL, GP/L: 2 × 10^3^ copies/mL), with a Ct value close to the declared lower limit (1 × 10^3^ copies/mL) of several kits, was detected by 17 participants. In addition, target sequences of other viruses (No. 1408 with HCV and MV VLP) did not produce cross-reactivity with the EBOV fragments.

We next evaluated the performance of different assays and participants. The commercially available assay from Daan Gene was the most frequently used and produced 100% accurate results at five agencies. Several participants selected the kits manufactured by Shanghai ZJ Bio-Tech Co., Ltd., one of which failed to detect the weakly positive specimens (No. 1405 and No. 1410). The primers offered by the Chinese CDC were selected by two participants, which achieved “competent” results. One participant used the RT-PCR and agarose gel electrophoresis method, and returned qualitative results (100% accurate rate) without a Ct value. However, another used an assay developed in-house and achieved only 30% accuracy, failing to detect any positive specimens. Additionally, no significant differences (*P* = 0.179) were observed between results obtained using commercial kits and those obtained using assays developed in-house.

## Discussion

From 2014 to 2015, the number of people suffering from Ebola hemorrhagic fever (EHF) increased continually in West Africa [13], and at one point the situation was out of control [14]. As the epidemic attracted international concern, many countries provided the required support for Ebola-affected areas. A Chinese medical team and public health experts [15] were assigned to the worst-hit countries, such as Sierra Leone, Liberia, and Guinea [16, 17]. Along with these personnel, several commercial real-time RT-PCR assays, including the kits manufactured by Daan Gene, ZJ Bio-Tech, Puruikang, Huada BioTech, and Tianlong, were used in EBOV detection in West Africa; these kits were also assessed in this EQA. Additionally, the assay manufactured by ZJ BioTech had attained the certification both from the Conformite Europeenne (CE) and WHO. The medical staff who worked in Ebola-affected regions and then returned to China needed to be isolated and to be examined for infection after a certain period of time. Given the presence of high-risk groups among Chinese citizens, adequate capacity for detection of and emergency response to EBOV had vital significance not only for China itself, but also for plague prevention at a global scale. Consequently, it is necessary to conduct this EQA for Ebola detection in China.

The majority of laboratories and commercial assays (73.68%) tested could detect EBOV with 100% accuracy. Notably, all negative specimens were identified correctly. However, efforts should be made to improve detection sensitivity. Apart from the seven positive samples that were not detected by the “improvable” participants, five positive samples were not detected. The conditions of the false-negative results should be considered carefully, to avoid errors in detection of weakly positive samples. In clinical practice, false-negative results could cause misdiagnosis and delay timely treatment and isolation of patients. In fact, PCR results are often negative at 2 or 3 days after the onset of clinical symptoms [18]. The earlier EBOV is diagnosed, the more timely measures can be taken, which has advantages for the control and prevention of EBOV.

In this assessment, we confirmed the dilution buffer composed of 30% normal human serum and 70% normal saline as sample matrix. In fact, three kinds of different proportions of dilution buffers (100% normal human serum, 30% normal human serum and 70% normal saline and 100% normal saline) were detected with the EBOV MS2 VLPs at the concentration of 10^5^copies/mL before finally determined. It was regarded as no difference because the variations among three types of dilution buffer were all less than 0.5 log_10_ copies/mL. In the dilution buffer, we mixed human serum for simulated EBOV clinical specimens and considering the influence of sample matrix in nucleic acid detection. Meanwhile, the stability of samples in the confirmed dilution buffer was further proved. Hence, it was feasible and rational for the sample panel with 30% normal human serum and 70% normal saline as dilution buffer.

The effects of different detection targets and methods should be further considered. Among the 19 participating agencies, Puruikang Bio-Tech Co., Ltd. and two provincial CDC offices used the GP/L site for detection, whereas the others chose the NP site. However, no significant differences (*P* = 0.462) in detection were found between the NP and GP/L sites. With respect to the various detection methods, the procedures for nucleic acid extraction and qualitative detection should be considered separately. First, we compared the paramagnetic particle and spin column methods for RNA extraction. Of the five participants that reported erroneous results, three used paramagnetic particle methods and two used spin column methods. No significant differences (*P =* 0.767) in overall detection were observed between the two methods for RNA extraction.

Second, the qualitative detection assays should be mentioned. Commercial detection assays were employed by 15 of the 19 participants, with an accuracy rate of 96.67%. Among them, the kits from Daan Gene and ZJ Bio-Tech were chosen with high frequency. Due to the small number of participating agencies, deep analysis of the various commercial reagents could not be conducted. In-house-developed real-time PCR assays using primers from the China CDC were adopted with 100% accuracy. Although institutions using common RT-PCR also reported 100% accurate results, this is not recommended, due to the importance of rapid diagnosis for EBOV control [14]. Notably, one participant failed to detect any of the positive samples in this evaluation. The primers used by this participant were designed for multiple types of EBOV, which likely decreased their analytic capability for single-type detection. For further identification of problems with detection, internal controls should be used, to exclude faults in the nucleic acid extraction process. The use of conventional and sensitive primers and probes for EBOV diagnosis is recommended.

Further improvement and optimization should be implemented, based on the feedback provided here. First, normalized detection assays provide accurate EBOV diagnosis. Commercial assays and the standard primers provided by the Chinese CDC were shown to be most trustworthy. Additionally, for all testing laboratories, it is advisable to implement a two-target assay approach, which could decrease the probability of false-negative results for viral mutations. Second, standardization of operations and punctual adjustment of instruments are also indispensable. In summary, effective and reliable EBOV detection requires recognition of problems and corresponding amendment of methods.

## Supporting Information

S1 Fig Verification of EBOV VLPs. (DOC)

S2 Fig Comparison of sample panels stored at -80°C and through blind mail. (DOC)

S1 Table Primers used in the present study. (DOC)

S2 Table The results of samples stability. (DOC)
