# Correction: External Quality Assessment of Molecular Detection of Ebola Virus in China

**DOI:** 10.1371/journal.pone.0151695

**Published:** 2016-03-10

**Authors:** Guojing Wang, Yu Sun, Kuo Zhang, Tingting Jia, Mingju Hao, Dong Zhang, Le Chang, Lei Zhang, Rui Zhang, Guigao Lin, Rongxue Peng, Jinming Li

S1 Fig and S2 Fig appear incorrectly in the published article. Please see the correct S1 Fig, S2 Fig, and their legends below.

## Supporting Information

S1 Fig Verification of EBOV VLPs. (A) Protein purification of EBOV MS2 VLP. Proteins of various molecular weights were differentiated by gel exclusion chromatography. The target protein of EBOV VLP was obtained around 60mins. (B) The visual results of EBOV VLPs in SDS-polyacrylamide gel electrophoresis. The bands between 10KD and 15KD were EBOV VLPs. Lane M, prestained protein molecular weight marker; Lane 1, EBOV VLP with NP fragments; Lane 2, EBOV VLP with GP/L fragments. (C) Enzymatic resistance of EBOV VLPs. After treated with RNase A and DNase I at 37°C, the single stripe over 1kb of 1% agarose gel electrophoresis proved the enzymatic resistance of VLPs encapsulated with EBOV fragments. Lane M, DL2000 DNA marker; Lane 1, EBOV VLP of NP fragment without incubated with RNase A and DNase I; Lane 2, EBOV VLP of NP fragment incubated with RNase A and DNase I; Lane 3, EBOV VLP of GP/L fragment without incubated with RNase A and DNase I; Lane 4, EBOV VLP of GP/L fragment incubated with RNase A and DNase I. (D) Reverse transcription PCR (RT-PCR) of the segments extracted from EBOV VLPs. After extraction of target EBOV nucleotides fragments encapsulated in EBOV VLPs, RT-PCR and 1% agarose gel electrophoresis were done for identification. The target bands between 2kb to 2.5Kb served as the successful proofs. Lane M, DL15000 DNA marker; Lane 1, positive control for NP fragment; Lane 2, RT-PCR products of RNA extracted from EBOV VLP of NP fragment; Lane 3, positive control for GP/L fragment; Lane 4, RT-PCR products of RNA extracted from EBOV VLP of GP/L fragment; Lane 5 and 6, negative controls.
(TIF)

S2 Fig Comparison of sample panels stored at -80°C and through blind mail. (A) The results of EBOV VLP with NP fragment. (B) The results of EBOV VLP with GP/L fragment. In both (A) and (B), all of the samples in panel from 1401 to 1410 were included. In order to distinguish different two sample panels, “*” was marked to the samples from blind mail. For each sample, three duplications were conducted and the distributions were revealed in scatter plot.
(TIF)
