# Supplementary material for: External Quality Assessment of Molecular Detection of Ebola Virus in China
Source: PLoS One. 2015 Jul 15;10(7):e0132659. doi: 10.1371/journal.pone.0132659 (PMC4503447; doi:10.1371/journal.pone.0132659)
Supplement: S1 Fig — (DOC) [file pone.0132659.s001.doc]

**Supporting Information**

**S1 Fig. Verification of EBOV VLPs.**

(A) Protein purification of EBOV MS2 VLP. Proteins of various molecular weights were differentiated by gel exclusion chromatography. The target protein of EBOV VLP was obtained around 60mins. (B) The visual results of EBOV VLPs in SDS-polyacrylamide gelelectrophoresis. The bands between 10KD and 15KD were EBOV VLPs. Lane M, prestained protein molecular weight marker; Lane 1, EBOV VLP with NP fragments; Lane 2, EBOV VLP with GP/L fragments. (C) Enzymatic resistance of EBOV VLPs. After digested with RNase A and DNase I at 37 ℃, 1% agarose gel electrophoresis with the single stripe over 1kb was used to prove the enzymatic resistance of VLPs encapsulated with EBOV fragments. Lane M, DL2000 DNA marker; Lane 1, EBOV VLP of NP fragment without incubated with RNase A and DNase I; Lane 2, EBOV VLP of NP fragment incubated with RNase A and DNase I; Lane 3, EBOV VLP of GP/L fragment without incubated with RNase A and DNase I; Lane 4, EBOV VLP of GP/L fragment incubated with RNase A and DNase I. (D) Reverse transcription PCR (RT-PCR) of the segments extracted from EBOV VLPs. After extraction of target EBOV nucleotides fragments encapsulated in EBOV VLPs, RT-PCR and 1% agarose gel electrophoresis were done for identification. The target bands between 2kb to 2.5Kb served as the successful proofs. Lane M, DL15000 DNA marker; Lane 1, positive control for NP fragment; Lane 2, RT-PCR products of RNA extracted from EBOV VLP of NP fragment; Lane 3, positive control for GP/L fragment; Lane 4, RT-PCR products of RNA extracted from EBOV VLP of GP/L fragment; Lane 5 and 6, negative controls.
