# Supplementary material for: External Quality Assessment of Molecular Detection of Ebola Virus in China
Source: PLoS One. 2015 Jul 15;10(7):e0132659. doi: 10.1371/journal.pone.0132659 (PMC4503447; doi:10.1371/journal.pone.0132659)
Supplement: S2 Fig — (DOC) [file pone.0132659.s002.doc]

**Supporting Information**

**S2 Fig. Comparison of sample panels stored at -80****°C and through blind mail.**

1. The results of EBOV VLP with NP fragment. **(B)** The results of EBOV VLP with GP/L fragment. In both (A) and (B), all of the samples in panel from 1401 to 1410 were included. In order to distinguish different two sample panels, “＊” was marked to the samples from blind mail. For each sample, three duplications were conducted and the [distribution](javascript:void(0);)swere revealed in scatter plot.
