# Supplementary material for: External Quality Assessment of Molecular Detection of Ebola Virus in China
Source: PLoS One. 2015 Jul 15;10(7):e0132659. doi: 10.1371/journal.pone.0132659 (PMC4503447; doi:10.1371/journal.pone.0132659)
Supplement: S1 Table — (DOC) [file pone.0132659.s003.doc]

**Supporting Information**

**S1 Table.** **Primers used in the present study**

| **Primer** | **Sequence (5’-3’)** |
| --- | --- |
| **NP ^a^-F** | CGGGGTACCACATGAGGATCACCCATGTCGGACACACAAAAAGAAAGAAGAATTTT |
| **NP ^a^-R** | CCTTAATTAAACATGGGTGATCCTCATGTTCATCTTGCGGGAG |
| **GP ^b^, L-F** | GGCAGATCT ACATGAGGATCACCCATGTAATCAACGAAGAGGCAGACCCACTG |
| **GP ^b^, L-R** | CGGGGTACCACATGGGTGATCCTCATGTATGACAATTATAAAAAACTTAATCTTAAAG |
| * Restriction sites are underlined. GGTACC: KpnI; TTAATTAA: Pac I; AGATCT: BglII.  ^a^ NP (segment 1): 3’ untranslated region (UTR), a large part of nucleoprotein (NP);  ^b^ GP, L (segment 2): the remaining part of NP, selected parts of GP, L and 5’ UTR. | |
