# Supplementary material for: External Quality Assessment of Molecular Detection of Ebola Virus in China
Source: PLoS One. 2015 Jul 15;10(7):e0132659. doi: 10.1371/journal.pone.0132659 (PMC4503447; doi:10.1371/journal.pone.0132659)
Supplement: S2 Table — (DOC) [file pone.0132659.s004.doc]

**Supporting Information**

**S2 Table.** **The results of samples stability**

|  | **The concentration of samples (copies/ml)** | | | |
| --- | --- | --- | --- | --- |
| **Temperature** | **10^5^** | | **10^3^** | |
|  | **NP** | **GP** | **NP** | **GP** |
| **RT** | 28.98±0.19 ^a^ | 23.47±0.19 | 35.87±0.30 | 29.56±0.44 |
| **4°C** | 28.76±0.19 | 23.55±0.13 | 35.48±0.47 | 30.18±0.27 |
| **-20°C** | 28.95±0.19 | 23.52±0.14 | 35.51±0.23 | 29.78±0.29 |
| **-80°C** | 29.03±0.03 | 23.41±0.09 | 35.58±0.11 | 29.82±0.25 |
| ^a^: The results of sample stability were showed in terms of mean Ct ± SD. | | | | |
